# Supplementary material for: HIV stigma and other barriers to COVID-19 vaccine uptake among Georgian people living with HIV/AIDS: A mixed-methods study
Source: PLOS Glob Public Health. 2024 Mar 28;4(3):e0003069. doi: 10.1371/journal.pgph.0003069 (PMC10977874; doi:10.1371/journal.pgph.0003069)
Supplement: Attachment — Submitted filename: Response to Reviewers.docx [file pgph.0003069.s002.docx]

**Response to Reviewers**

***Reviewer #1:***

| # | Review Comments to the Author | Response to the comments |
| --- | --- | --- |
| 1 | Line 26: “Between December-July 2022” State the year of commencement i.e December 2020, December 2021 e.t.c | Added “December 2021-July-2022” |
| 2 | Line 46: Remove “and death” | “and death” removed |
| 3 | Line 77: Change “not” to “no” | Changed “not” to “no” |
| 4 | Line 143: Please, mention the specific type of regression used i.e binary, multinomial e.t.c | Revised accordingly in the Statistical Analysis section. |
| 5 | A brief description of the study area should also be made. | Added in the methods section: “The study was conducted throughout Georgia, covering the capital city, Tbilisi, as well as various regions, including Kakheti, Imereti, Guria, Samegrelo, and Ajara. Utilizing different online meeting options (e.g. Zoom, WhatsApp, Viber) during the data collection facilitated interviews with participants from diverse geographical locations.”) |
| 6 | Sample size estimations should be presented. Considering inferential statistics were conducted this is mandatory | Thank you for this important comment. Added to the statistical analysis section accordingly: “OpenEpi v3.03, an open-source tool, was employed to determine the sample size for this study. With a 5% margin of error and a 95% confidence interval, and considering a population size of 9,400, as estimated by the World Health Organization for people living with HIV in Georgia in 2018, the recommended sample size was determined to be 370. This calculation assumed a 50% response distribution.”) |

***Reviewer #2:***

| # | Review Comments to the Author | Response to the comments |
| --- | --- | --- |
| 1 | Line 63, are the authors referring to “being immune to COVID-19” OR “…being immune with COVID-19 vaccine hesitancy” | Authors are referring to “being immune to COVID-19”. The sentence was revised accordingly: “The study conducted among PLWH in France reported association between a general vaccine refusal, fear of developing side effects and perception of already being immune with COVID-19 and COVID-19 vaccination hesitancy”) |
| 2 | It is good that the authors have provided information on the lack of data on vaccine hesitancy among PLHIV in EECA region. However, the flow of the text on P3-4, L64-73 seems to be disjointed. It could be improved by rephrasing and restructuring the sentences. The authors may consider integrating those sentences into one paragraph. Also, it is worthy to note that the attitude observed in the general population may not be reflected among PLHIV. | Introduction section has been updated according to the title change as suggested by reviewer 3. |
| 3 | P4, L74 consider changing “There are not data” to “there is no data” | “There are not data” changed to “there is no data” |
| 4 | The authors highlighted that the COVID-19 vaccine uptake rates in Georgia was suboptimal. One will wonder whether this is associated with availability and accessibility of the vaccine in the country. And to make a fairer comparison with other nations one would like to know when the rollout was commenced in the country. The authors should also consider obtaining an updated vaccination uptake rates in the country, as the data might have changed between July 2022 to date. | Thank you for this valuable comment. Information about vaccination commencement added “Georgia received the first batch of COVID-19 vaccine in March 2021”.  As for the updating statistics, the data presented in the manuscript are the latest available data for Georgia. No updates have been reported after that period. |
| 5 | The sentence that started with “The other important factors….” in L81-83 is not well fitted in that position. According to my understanding, the paragraph was intended to provide justification for conducting the study. Can this sentence be moved to where they discussed the factors associated with vaccine acceptance/hesitancy in other parts of the introduction? This will improve the flow and coherence of the text. | Introduction section has been updated according to the title change as suggested by reviewer 3. |
| 6 | Considering the study design, it will be more transparent and appropriate if the authors can provide justification for their sample size. | Sample size estimation was added in the statistical analysis section. And actual sample size justification added to the results section: “We observed a considerably low participation rates (85 enrolled vs 370 planned) in our study. This was one of the first studies among PLWH that used online interviewing, which might result in a low response rate. Another factor contributing to low participation rates could be accounted to widespread and deeply rooted stigma towards HIV in Georgia, which could prevent PLWH’s participation in the study (even though PLWH community-based organization supported the recruitment). Another factor for consideration is the time frame of the study, which did not allow to further continue enrolment of study participants.” |
| 7 | The number of participants given in the caption of table 1 (L184) does not correspond to the total number presented on the table. Similarly, it seems the percentage mentioned in L188 in not correct. Additionally, the authors may consider introducing subheadings in the results section to improve the readability. | The numbers and percentages corrected accordingly. |
| 8 | One will wonder why the authors omitted many important sociodemographic characteristics (such as ethnicity/race, employment status, income etc.) and factors related to the HIV itself (utilization of ART, virologic suppression, etc.) and the COVID-19 vaccine (in the questionnaire), which have been proven to be essential in influencing vaccine acceptance and hesitancy. These would have provided more detailed information about the study population. In addition, have the authors attempted to verify the information provided by the participants with their medical record, especially those related to the vaccination? It is also relevant to include the number doses received by the vaccinated group. | Race is not relevant for Georgia.  In terms of income, we asked about their financial situation over the past 3 months, whether it has improved, worsened or stayed the same.  Due to constraints in questionnaire length and the need to maintain participant engagement, certain demographic variables, including employment status, utilization of ART, virologic suppression were intentionally excluded to streamline the survey and ensure a more focused examination of other key determinants.  Accessing and cross-referencing individual medical records are time-consuming, costly, and require additional permissions, thus in the planning process, the decision was made not to verify self-reported data with participants' medical records. |
| 9 | The discussion section of the manuscript needs to be improved. I suggest comparing the vaccination rate obtained in this study with what was reported in other parts of the globe among the same population. Also, comparison should be made with the latest data among the general population in the same country. Moreover, since stigma is key aspect of the study, it is important to further support this finding with the available evidence (if any) on the role of stigma on vaccine acceptance/hesitancy. | Thank you for this important comment. The discussion section updated. Relevant comparisons on vaccine uptake included and evidence on the role of stigma added. |

***Reviewer 3***

| # | Review Comments to the Author | Response to the comments |
| --- | --- | --- |
| 1 | Lines 65-66 the authors, without supporting with a reference wrote: “Data for EECA indicates that HIV epidemic continues 66 to grow in the region” | Reference added. |
| 2 | In Table 1 title, the authors wrote “…..characteristics of PLWH (n=84) and COVID-19 experience”. Is the n = 84 or 85 | 84 changed to 85. |
| 3 | In Table 2, the authors wrote “What do you think how can be handled the COVID-19 situation”. Please rephrase for clarity. | Rephrased: “How can the COVID-19 situation be handled through government policies?” |
| 4 | What approach to logistic regression modeling have the authors used? Forward, backward, or stepwise?. | Added to the statistical analysis section: “To build the model, we used stepwise regression. First, we evaluated all variables for their unique contribution in the model. Second, if one did not contribute, we removed it from the model. However, we reentered it later, if this variable was able to explain a bit more significant variance in the dependent variable than it did when it first came in.” |
| 5 | Why have the authors not provided the full results of the bivariate and multivariate regression analysis. Please provide this for transparency even if no significant association is found it is still important. | For bivariate analyses the results are provided in table 3. As for the multivariable analyses, the best models were chosen based using stepwise regression. In addition, Akaike Information Criterion was used to assess model fit statistics. |
| 6 | The authors have provided data on important behavioural determinants of vaccination in PLHIV but have not included most of these in their regression analysis. Examples include: I will not have a severe form; Health care providers are not friendly at vaccination place; He/she may need disclose my HIV status and he/she may not want it; I have lost friends by telling them I have HIV; I am very careful who I tell that I have HIV; and overall COVID-19 knowledge and attitude. Please report the results of all analysis in full even as a supplementary information. | As we mentioned earlier, for the multivariable analyses, Co-variates identified by a directed acyclic graph and/or co-variates with a p-value <0.05 in the bivariate analysis were included in the multivariable analysis. Best models were chosen based using stepwise regression. In addition, Akaike Information Criterion was used to assess model fit statistics.  Please, note that “I will not have a severe form” is included in the in the bivariate analysis and in the multivariable model as the first exposure (see the table 4). As for other behavioral determinants, (such as Health care providers are not friendly at vaccination place; He/she may need disclose my HIV status and he/she may not want it; I have lost friends by telling them I have HIV; I am very careful who I tell that I have HIV; and overall COVID-19 knowledge and attitude), the bivariate analysis that included all mentioned determinants was conducted and results are reported in the Table 3. However, the bivariate association of outcome of interest with these variables was not significant (see Table 3) and therefore, they were not included in the multivariable regression models. |
| 7 | Also 45 out of the 85 participants have been vaccinated according to the authors, while 40 were unvaccinated. It should be noted that these 40 unvaccinated participants can not be said to be hesitant (unless you’ve asked them and they all said they didn’t intend to vaccinate, which you’ve not stated anywhere in your manuscript) according to the WHO SAGE Working group of experts on vaccine hesitancy (delay in acceptance or 51 refusal of vaccination despite the availability of vaccination services) stated and cited by the authors in the introduction. I am saying this because the word hesitancy is included in your manuscript title although not anywhere in the results section. Therefore, the authors need to rephrase the article title and remove the word “hesitancy” from it because its not what they evaluated. Similarly, they should apply this same change to all relevant sections of the manuscript especially the discussion section. | Thank you for this very important comment.  The article title rephrased: “HIV stigma and other barriers to COVID-19 vaccine uptake among Georgian people living with HIV/AIDS: a mixed-methods study”.  Similar changes applied to all relevant sections of the manuscript. |
| 8 | Despite only evaluating the determinants of COVID-19 vaccine uptake, the authors kept citing studies on determinants of hesitancy and acceptance in their study. The authors may wish to consider this important manuscript for guide: Sulaiman, S. K., Musa, M. S., Tsiga-Ahmed, F. I. I., Sulaiman, A. K., & Bako, A. T. (2023). A systematic review and meta-analysis of the global prevalence and determinants of COVID-19 vaccine acceptance and uptake in people living with HIV. Nature Human Behaviour, 1-15. <https://doi.org/10.1038/s41562-023-01733-3>. | Thank you for the valuable suggestion. It has been considered accordingly. |
